# Wind speed, sun exposure and water status alter sunburn susceptibility of grape berries

**DOI:** 10.3389/fpls.2023.1145274

**Published:** 2023-03-27

**Authors:** Kai Müller, Markus Keller, Manfred Stoll, Matthias Friedel

**Affiliations:** ^1^ Department of General and Organic Viticulture, Hochschule Geisenheim University, Geisenheim, Germany; ^2^ Irrigated Agriculture Research and Extension Center, Washington State University, Prosser, WA, United States

**Keywords:** *Vitis vinifera*, sunburn, drought-stress, heat stress, fruit surface temperature, wind speed

## Abstract

In the context of climate change, yield and quality losses from sunburn necrosis are challenging grape growers around the world. In a previous review, we identified the role of wind speed, duration of heat exposure, drought stress and adaptation as major knowledge gaps that prevent a better predictability of sunburn events. In this paper we present results of targeted experiments aiming to close these knowledge gaps. The effects of drought stress and adaptation on sunburn susceptibility were investigated in a combined drought stress/ defoliation experiment. Riesling grapevines growing in an arid climate were fully irrigated or drought stressed, and clusters were exposed to sunlight by fruit-zone leaf removal (defoliation) at two developmental stages. Sunburn symptoms were induced using infrared heaters while fruit surface temperature was measured using thermal imaging enabling the establishment of threshold temperatures. The influence of the duration of heat exposure of berries was examined by heating grape clusters to a stable temperature and monitoring the evolution of sunburn symptoms over time. To examine the effects of wind speed on the appearance of sunburn necrosis symptoms, fruit surface temperatures and sunburn severity were measured along an artificially induced wind speed gradient in two cultivars using thermal imaging and visual inspection. Longer durations of heat exposure required lower fruit surface temperatures to induce damage, while the differences in temperature after 60 min and 90 min of exposure were marginal (47.82 ± 0.25 °C and 47.06 ± 0.26 °C). Clusters of vines grown under water deficit were less susceptible to sunburn compared to those of well-irrigated plants following defoliation. The lethal temperature of clusters exposed to sunlight for seven days did not differ from those exposed to sunlight for 28 days, indicating that a full adaptation ocurred within this period. Higher wind speeds led to lower cluster temperatures and reduced sunburn severity. First evidence of a drought priming induced heat tolerance of grapevine berries was found, while adaptation had a more pronounced effect on the susceptibility to sunburn compared to water stress.

## Introduction

In the context of climate change, grapevines have to cope with higher temperatures and lower soil water availability during the vegetation period (Schultz, 2019; Santos et al., 2020). Heat load during key phenophases is projected to rise not only due to rising temperatures, but also due to an acceleration of phenological development (Yang et al., 2022). In addition, more intense, more frequent and longer lasting heatwaves during key phenophases are expected to occur (Meehl and Tebaldi, 2004). This can result in substantial yield losses due to sunburn necrosis (SN) or in a loss of crop value due to sunburn browning (Gambetta et al., 2021). Water scarcity is arguably the most critical threat to agricultural productivity and in many important winegrowing regions as climate change intensifies. Drought might exacerbate adverse effects of heat stress by increasing canopy temperatures through reduced transpiration (Schrader et al., 2003). Higher yield losses were observed following a heatwave in vineyards that concurrently suffered from drought stress in Australia (Webb et al., 2010).

The berry temperature plays a key role in the development of SN symptoms. Berry temperature is mostly a function of the degree of exposure to direct sunlight, as the absorption of radiation may cause berry temperatures to rise well above ambient (Smart, 1985; Gambetta et al., 2021). The ability to adapt to heat stress and to acquire thermotolerance during phases of sublethal heat exposure has been shown for various fruit species, including grapevine (Morales-Quintana et al., 2020; Gambetta et al., 2021). Sublethal stress was shown to enhance the transcription of several heat shock factors and heat shock proteins that are important for acquired thermotolerance (Morales-Quintana et al., 2020). Berries growing under elevated temperatures accumulated heat shock factors that are associated with the formation of molecules that mediate heat stress in cells (Pillet et al., 2012).

Sun-exposed berries may adapt to high light and temperature exposure by forming a thicker epidermis which increases the amount of photoprotective pigments like chlorophylls, carotenoids or phenolic compounds that are related to stress mitigation. The formation of a more plate-like and thicker epicuticular wax layer under these conditions may additionally reflect radiation more efficiently (Gambetta et al., 2021). To increase the accumulation of quality-related compounds, improve spray coverage of clusters for disease control, and accelerate the drying of clusters after rain events, partial or total defoliation of the cluster zone has become a popular practice in viticulture (Smart, 1985; Reynolds and Vanden Heuvel, 2009).

When performing partial defoliation during a period of relatively mild temperatures, grape berries may experience a sublethal stress through elevated temperatures that do not lead to injuries (Morales-Quintana et al., 2020). The same stimulus, if performed during a period of excessive temperatures causes lethal damage of the cells and hence leads to SN or to sunburn browning (Lopes et al., 2019).

Due to an alteration in gas exchange and growth rate, water deficit increases canopy porosity and sun exposure, and hence the temperature of the fruit (Keller et al., 2016). Even though water stress may be a limiting factor for natural shading of the clusters by the canopy, the acquisition of thermotolerance might be enhanced *via* cross-priming reactions induced by drought stress (Morales-Quintana et al., 2020). Drought stress promotes the accumulation of some secondary metabolites that prevent oxidative damage, including carotenoids in white cultivars or anthocyanins in red cultivars (Gambetta et al., 2020). An increase in flavonols observed in berries of water stressed vines is more likely induced due to a higher light exposure (Torres et al., 2021).

Apart from absorbed radiation and ambient temperature, wind speed is a key determinant of berry temperature (Smart and Sinclair, 1976). By reducing the boundary layer resistance and thus increasing surface heat loss *via* forced convection, higher wind speed may have the potential to prevent heat damage. In a field trial, the wind speeds within a defoliated canopy were consistently higher compared to a non-defoliated canopy (Thomas et al., 1988), which could attenuate the effects of higher sunlight exposure to some degree.

The aim of this study was to address knowledge gaps around heat damage formation in grapes to make sunburn events more predictable and thus manageable. We hypothesized that berries are able to enhance their resilience to SN damage under water stress conditions and light exposure treatments by leaf removal and that altered wind speeds are able to reduce SN severity. To this end, we examined possible interactions of drought stress, the timing of leaf removal, and the developmental stage on the susceptibility of grape berries to sunburn by implementing a method to investigate differences in sunburn susceptibility as described in Müller et al. (2022) for *Vitis vinifera* L. cv. Riesling, one of the major white grape cultivars worldwide. The influence of the duration of heat exposure on the occurrence of SN symptoms and the influence of wind speed on berry temperature and SN development were tested in two additional experiments.

## Materials and methods

### Irrigation and defoliation experiment

The experiment was conducted in 2022 in a vineyard planted in 2010 at the Irrigated Agriculture Research and Extension Center (46.29°N; 119.73°W; 345 m a.s.l.) near Prosser, Washington, USA. In this arid climate zone in the Yakima Valley the mean annual precipitation is ~200 mm with average annual temperature of 12°C, average growing season (April-October) temperature of 16.5°C, and average seasonal growing degree days (GDD > 10°C) of 1400°Cd. The rows planted with *V. vinifera* cv. Riesling (clone FPS 09, own-rooted) were north-south orientated. Vines were trained to vertical shoot positioning (VSP) and spaced 1.82 m apart with row spacing of 2.74 m. The soil is a Warden silt loam with a pH of 8.0 and 26% (v/v) soil moisture at field capacity and 8% at the permanent wilting point.

The field experiment included two irrigation treatments (main plots) with four rows each and three defoliation treatments (sub-plots, early defoliation (ED, EL-27, 10 days after fruit set), late defoliation (LD, EL-32, 25 days after fruit set) and a non-defoliated control (ND)), in a split-plot design with five replicate blocks. Only the fruit zone on the east side of the canopy was defoliated to avoid excessive heat damage on the clusters. Two to three leaves were removed manually from the base of each shoot so that the clusters were exposed to sunlight during the morning hours. (sub-plots). Each replicate block consisted of 6 vines, whereas the two outer vines served as a buffer to the neighboring blocks. A full irrigation treatment (FI, no water stress) and a drought stress treatment (DS) was established using drip irrigation. Vines were irrigated weekly to a target soil moisture of 16% and a vine water status, measured as midday leaf water potential, ranging from -0.8 to -1.0 MPa. DS vines were not irrigated from 13 June to 08 August (EL-27 to EL-35). Before budbreak and after harvest, soil water was replenished to field capacity to avoid water stress before bloom and during winter.

Monitoring for SN was done prior to harvest (23 September) with 25 clusters per block being evaluated on both sides of the canopy. SN was monitored on a free scale from 0-100 as a percentage of damage of the whole cluster.

Temperature (°C) and light intensity (lx) sensors (HOBO Pendant Data Logger, Onset Computer Corporation, Bourne, MA, USA) were placed next to representative clusters on the east and west side of the canopy. The sensors were set up in the DS treatment to evaluate differences in the defoliation treatments. Data was collected in 30 min intervals starting from day of year (DOY) 192, approximately 14 days after fruit set and 4 days after ED was applied. The dataloggers on the west side of the canopy and those in non-defoliated treatments were placed underneath a layer of leaves in the fruit zone, and their correct positioning was periodically monitored. Temperatures were calculated as mean temperatures per hour with two measurements per hour. During the heating experiments (described below), some of the sensors of the non-defoliated controls were exposed due to partial defoliation; their measurements were excluded from the data set from that timepoint forward. Temperature (°C) and solar radiation (W m^-^²) values at 2 m height were measured at hourly intervals by a weather station located at the north-east corner of the vineyard.

Sunburn was induced by heat treatment using the method described by Müller et al. (2022). Briefly, grape clusters were heated to a target temperature using infrared lamps and temperature sensors inserted in the center of the clusters. The power of the lamps was regulated by a control unit, which continuously read the actual temperature given by the sensors and altered the power of the lamps using a control algorithm. Six lamps were controlled individually by the control unit consisting of a single-board computer connected to a power stage with phase control modulator and a touchscreen for manual operation.

Sunburn was induced by heating at two developmental stages: berries at pea-size (EL-31, 19 and 20 July) and lag phase (EL-33, 13 and 14 August) for each of the three defoliation treatments. The first sunburn induction occurred 7 days after ED, and the second sunburn induction occurred 7 days after LD. Immediately before the sunburn induction, the fruit zones of treated vines and non-treated control vines were defoliated to ensure that sunlight reached the clusters during the heat treatment. Six clusters per replicate were heated to a sensor target temperature of 48°C for 30 min. The distance of single berries to the center of the lamp, their position in the cluster and thus their distance to the sensor alters their recorded surface temperature. This yields a range of berry temperatures along the cluster which is necessary for the calculation of a lethal temperature. Midday leaf water potential (Ψ_L_) was measured between 13:00 and 14:00 PST around the days of the experiments for each vine of the experiment. One mid-sized leaf per vine, approximately 5-6 nodes from the top of the shoot was selected and enclosed in a plastic bag before excision. The measurements were performed with a pressure chamber (model 615D, PMS Instrument Company, Albany, OR, USA).

The fruit surface temperature (FST) of single berries was measured using infrared thermography. A thermal image of the cluster was taken using an infrared camera (model: H2640, Nippon Avionics Co., Ltd., Yokohama, Japan). Thermograms were analyzed with the InfReC Analyzer NS9500 LT software (Version 5.0C, Nippon Avionics Co., Ltd., Yokohama, Japan). Mean FST of an individual berry was calculated as the mean temperature of all pixels of a manually selected circular area following the shape of the berries on the thermogram.

### Heat duration experiment

The effect of the duration of heat applied to grape berries on their susceptibility to sunburn was examined at an early developmental stage (pea size, EL-31). Six clusters on two vines each were heated to a target temperature of either 46°C or 48°C without direct sunlight on the clusters. Thermal and digital images of each cluster were taken before the start of the heating and then after 15, 30, 60 and 90 min. To take thermal images at the same timepoints, heating of each cluster started with a delay of 60 sec from the start of the heating of the previous cluster. For each cluster, the mean FST of all visible berries was calculated so that each berry was repeatedly measured for each timepoint. Each berry was classified as either symptomatic or non-symptomatic for SN by visually examining the digital images from each timepoint. The surface temperatures were calculated as mean temperatures from the beginning of the experiment to each point of measurement by adding the previous measured temperatures and dividing the sum by the time that had passed. Berries that were annotated as symptomatic were excluded from the dataset for the subsequent timepoints.

### Wind experiment

The experiment was conducted on 19 July 2022 in a vineyard planted in 2015 at Hochschule Geisenheim University (49.99°N; 7.95°E, 110 m a.s.l.). The region has a mean annual precipitation of ~540 mm with average annual temperature of 11°C, average growing season (April-October) temperature of 15.7°C (1991-2020). Rows were alternately planted with *V. vinifera* cv. Riesling (clone 365 grafted to SO4 rootstock) and Calardis Blanc ((Bacchus x Seyval Blanc) x Seyve Villard 39-639 grafted to SO4 rootstock) and oriented north-south. From a breeder´s perspective, Calardis blanc compared to Riesling can be considered as resilient to sunburn, even in very dry and hot years (JKI, 2023). Vines were cane-pruned to a single cane and trained to a VSP system and spaced 1.2 m apart with a row spacing of 2 m. The soil is a silty loam with an available field capacity (2 m) of 300 mm or 15% (v/v).

At the time of the experiment, both cultivars were at phenological stage EL-32. Before the experiment, 13 and 12 shaded clusters per cultivar were selected on a 3 m stretch of canopy and marked. The selected clusters were all located at a comparable height above ground (1.0 – 1.2 m). After solar noon (14:00 CET), all clusters were exposed to direct sunlight by leaf removal on the western side of the canopy. Although all leaves in the cluster zone were removed, clusters located on the eastern side of the canopy were not sun-exposed throughout the experiment, and thus a portion of clusters remained in canopy shade. Immediately after leaf removal, cluster temperatures were measured by infrared thermography. After the thermograms were taken, a wind gradient was applied along the cluster zone using column fans (DO1100E, Duracraft, Southborough, MA, USA). Wind speed along the 3 m stretch of canopy was measured by a hand-held anemometer (Agrotop, Obertraubling, Germany). The distance of all marked clusters from the fans was recorded. Thermograms were taken at 0, 10, 30, 60, 120 and 180 min and wind was maintained until the cluster zone was shaded by the adjacent rows (about 18:00 CET). Visible SN symptoms were documented on a scale from 0-100% the day after (9:00 CET) for all marked clusters and for all clusters in the 3 m canopy stretch, divided into 30 cm sections.

### Data analysis

R software (v. 4.0.3, R Core Team, 2020) was used for statistical analysis. Depending on the experiment, a binary logistic regression model was calculated with SN symptoms as the response variable and either ‘irrigation treatment’, ‘defoliation treatment’ and ‘developmental stage’, or ‘surface temperature’ and ‘heating time’ as predictors. A Wald chi-squared test (p < 0.05) was used to test the significance of the predictors. Using the *MASS* package, the lethal temperature for 50% of the berries (LT_50_) was calculated for each heating time (Venables et al., 2002). A linear model was calculated to predict LT_50_ based on heating time. Sunburn severity was tested for normal distribution using an Anderson-Darling test. The test was statistically significant (A = 1.268, p = < 0.001) indicating no compliance with the normality assumption. A linear mixed model ANOVA was performed prior to a pairwise comparison using Z-tests, corrected with Holm’s sequential Bonferroni procedure (p < 0.001).

## Results

### Heat duration experiment

**Figure 1:**
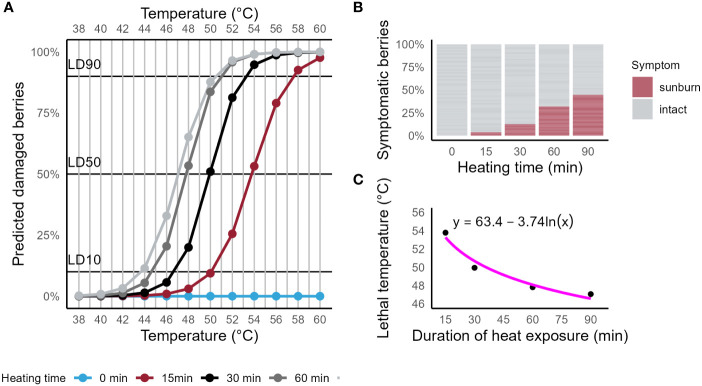
**(A)** Prediction plot for the occurrence of sunburn symptoms on Riesling berries exposed to high temperatures with periodic measurements of berry surface temperature. Binary logistic regression model showed significance for predictors *Surface temperature* (Wald = 123.66, p < 0.001) and *Time* (Wald = 163.62, p < 0.001). **(B)** Bar plot of the cumulative percentage of berries observed with symptoms of sunburn necrosis for four timepoints (0, 15, 30, 60 and 90 min). **(C)** Scatter plot of calculated lethal temperatures (LT_50_, probability for symptomatic berries = 50%) for four durations of high heat exposure of grape berries (15, 30, 60 and 90 min). A regression fit is represented by the solid line (R² = 0.94, F (1,2) = 46.48, p = 0.021).

**Figure 1A** shows the predicted probability of SN symptoms to appear after berries were exposed to high temperatures. The FST was measured at five timepoints throughout the heat exposure. A binary logistic regression model was used to calculate the contribution of the two predictors *Surface temperature* (Wald = 163.62, p < 0.001) and *Time* (Wald = 123.66, p < 0.001). For each 1°C increase in FST, the probability for berries to show SN symptoms was 1.007 times higher. Likewise, with an increase of 1 min of heat exposure, berries were 3.34 times more likely to show SN symptoms. Both effects are visualized by different slopes of the prediction curves and by the shift of each prediction curve on the abscissa. Symptomatic and non-symptomatic berries were determined for each timepoint and are visualized in **Figure 1B** as percentages of symptomatic berries.

With longer durations of heat exposure, lower FST were required for berries to show symptoms of SN. LT_50_s for the timepoints 15, 30, 60 and 90 min were 53.79 ± 1.10°C, 49.94 ± 0.41°C, 47.82 ± 0.25°C and 47.06 ± 0.26°C, respectively. The calculated LT_50_ predicted the duration of heat exposure required to induce damage at that temperature (R² = 0.94, F (1,2) = 46.48, p = 0.021) as visualized by the regression fit (solid line) in **Figure 1C**.

### Irrigation and defoliation experiment

**Figure 2:**
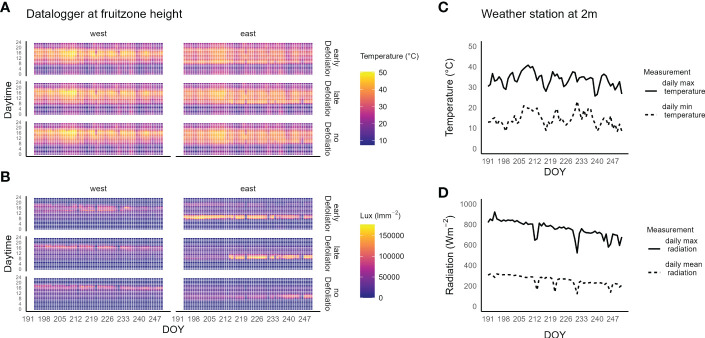
**(A)** Heatmap of the hourly mean temperature of Riesling grapes in a vineyard in southeastern Washington, USA. **(B)** Heatmap of the hourly mean light intensity. Measurements were taken on drought stressed vines at fruit-zone height on the east and west side of the canopy for three defoliation treatments (early defoliation, 10 days after fruit set, late defoliation, 25 days after fruit set and no defoliation). **(C)** Daily maximum (solid line) and daily minimum (dashed line) temperature measured at 2 m height at the weather station located next to the vineyard. **(D)** daily maximum (solid line) and mean (dashed line) global radiation.

In the irrigation and defoliation experiment conducted in Washington, the hourly mean temperatures, measured by dataloggers at cluster level, reached a maximum of about 50°C on the west side of the canopy (**Figure 2**). The highest temperatures were found on DOY 208 to 212, when the weather station registered about 40°C daily maximum temperatures. The ED, east-side cluster zone had a 1.5 ± 0.3°C cooler daily mean temperature compared to the ND cluster zones. The highest temperatures were found on the ND west-side of the canopy between 16:00 to 19:00 PST with 46.1°C mean temperature (**Suppl. Data 1**).

Midday leaf water potential was measured on two developmental stages prior to the heating (i.e., sunburn induction) experiments. In the FI block, the mean Ψ_L_ was -0.82 ± 0.07 MPa at EL-31 and -0.8 ± 0.09 MPa at EL-33. In the DS block, the mean Ψ_L_ was -1.28 ± 0.05 MPa at EL-31 and -1.34 ± 0.69 MPa at EL-33.

**Figure 3:**
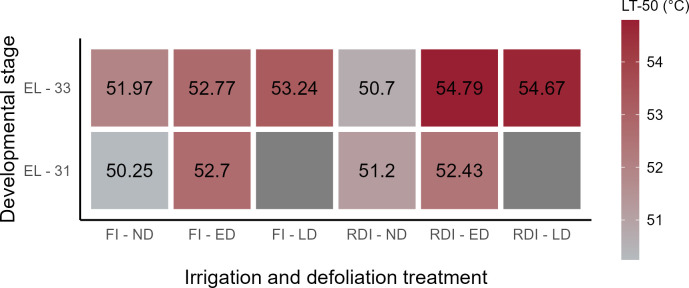
Heatmap of the predicted Lethal Temperature 50 (LT_50_, Temperature for 30 min of heat exposure at which the probability of SN symptoms to occur is 50%). The LT_50_ was calculated for two developmental stages (pea-size (EL-31) and lag-phase (EL-33)), two irrigation treatments (FI, fully irrigated; DS, drought-stressed) and three defoliation treatments (ND, not defoliated, ED; early defoliated; LD, late defoliated).

The LT_50_ in three defoliation treatments and in two irrigation plots was calculated for two developmental stages as shown in **Figure 3**. At EL-31, the LD treatments were not evaluated since they were not defoliated at that time, thus were congruent to the ND treatment. LT_50s_ were higher in ED and LD compared to ND for FI vines and likewise higher for DS at both developmental stages. While ED treatments at EL-31 had similar LT_50_ between FI and DS, the LT_50_ was 2°C higher at EL-33 with LD compared to ND following a similar trend. ED and LD were at a similar level at EL-33 for FI (-0.5°C) and DS (+0.1°C). The LT_50_ of the ND clusters in the DS plots was 1.2°C lower at EL-33 compared to ND clusters in the FI plots.

**Figure 4:**
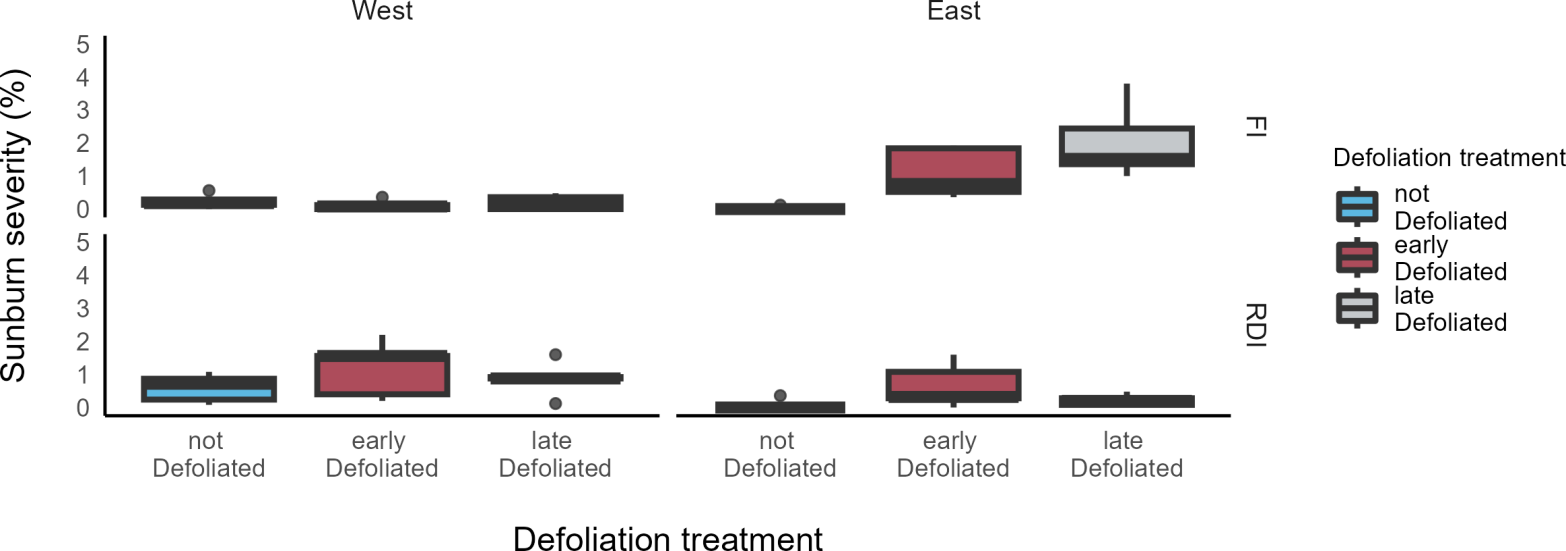
Boxplots of sunburn severity prior to harvest (23 September) in two irrigation treatments (FI, fully irrigated; DS, drought-stressed) and three defoliation treatments (ND, not defoliated; ED, early defoliated; LD, late defoliated) on both east and west side of the canopy in a vineyard in southeastern Washington.

The highest SN severities as shown in **Figure 4** were found in the defoliated cluster zone (east) of the ED and LD treatment in the FI plot, with no significant difference between both treatments (p = 0.09). On this canopy side, LD of the FI plot was significantly higher compared to LD of the DS plot (p < 0.001), while ED was not different between the irrigation plots (p > 0.05). Pairwise comparison across all DS defoliation treatments showed no significant differences. No significant differences in SN severity were found between the east side of ND treatments of both irrigation plots. Sunburn severity on the west side of the FI treatment was relatively low for all defoliation treatments with no significant differences between them or to the ND treatments at the east side of each irrigation plot (p > 0.05)

### Wind experiment

**Figure 5:**
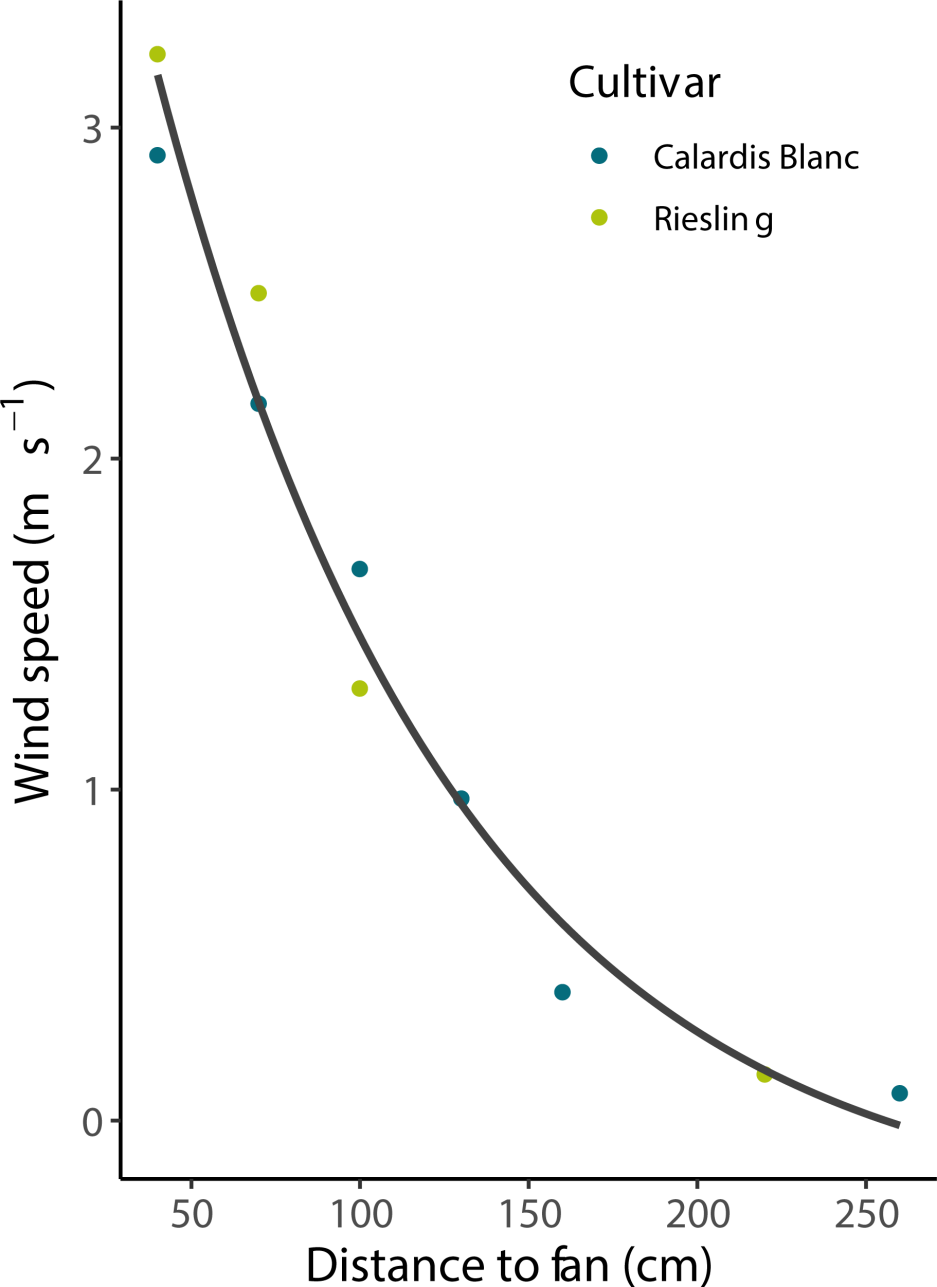
Wind speed as affected by the distance from the column fan. Line represents a second order polynomial fit that was used to predict wind speed at the location of individual clusters by measuring their distance to the column fan.

**Figure 6:**
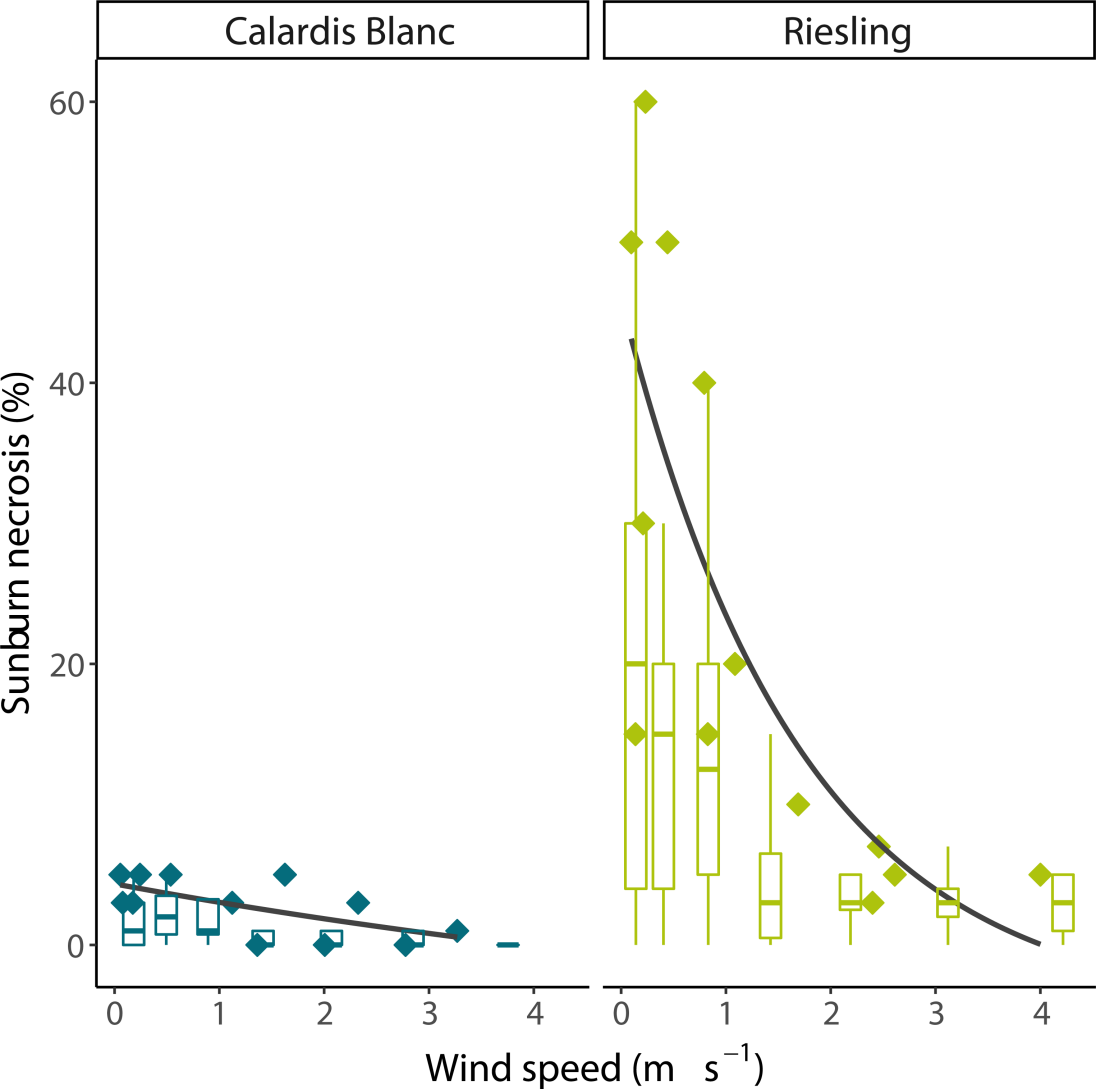
Effect of wind speed on sunburn necrosis severity of Calardis Blanc and Riesling grape clusters in a vineyard at Geisenheim. Box plots represent all clusters in the canopy, symbols represent marked, formerly shaded clusters. Solid lines represent logarithmic fits of necrosis severity of marked clusters vs. wind speed.

In the wind experiment, Calardis Blanc and Riesling showed a significantly different sunburn damage (p < 0.001, n = 108), with 0.8% and 8.9% severity, respectively, in the control (no wind) despite showing comparable berry temperatures. The wind speed gradients for both cultivars were comparable as shown in **Figure 5**, and measured wind speed ranged from 0.1 m s^-1^ to 3.25 m s^-1^ at 260 cm and 40 cm distance from the fan. Integrating the results across all clusters, it was shown that after 30 min, berry temperatures were stable along the wind gradient until the end of the experiment. Cluster temperature was significantly correlated to wind speed after stable temperatures were reached (p < 0.001, R² = 0.39, n = 140 bunch sections). The mean temperature of the clusters at 160-190 cm from the fan was about 3°C warmer than that of clusters at 20-40 cm distance (43.5 and 40.5°C, respectively). As visualized in **Figure 6**, the SN damage observed in the marked, previously shaded clusters correlated significantly with the wind speed predicted for the individual clusters in both cultivars (n = 13, p < 0.001 for Riesling and n = 12, p < 0.01 for Calardis Blanc, respectively). Sunburn damage in Riesling, however, occurred even at high wind speeds of 2.25-3.9 m s^-1^ in all previously shaded clusters, although the damage was lower than at low wind speeds. No cooling effect could be detected at a distance > 190 cm (wind speed ~0.3 m s^-1^) in either cultivar and damage severity was relatively uniform.

## Discussion

This study demonstrated that the duration of short-term exposure to high temperatures modulates the LT_50_ and provided evidence for priming and cross-priming effects that increase heat tolerance of grapevine berries after exposure to drought. It further demonstrated that wind decreases sunburn incidence and severity by reducing the temperature of sun-exposed clusters.

### Heat duration experiment

The probability of a berry to develop symptoms of sunburn necrosis depends on both intensity and duration of the heat that it receives. Berry surface temperatures of 50 ± 0.2°C led to a 10% probability for damage after 15 min, 50% probability after 30 min and close to 90% probability after 90 min of heat exposure. Other authors found symptoms of sunburn necrosis on apple peel after 10 min of heat exposure to 52.2°C with sunlight excluded and symptoms of sunburn browning after 1 h with temperatures of about 48°C with sunlight during the heating time (Schrader et al., 2001). Whilst the present study added to our understanding of the effects of duration and intensity of heat on the development of SN symptoms for grape berries, we did not observe any sunburn browning. Schrader et al. (2001) found symptoms of SN when apples were sunlit while being heated to a certain threshold, thus direct sun exposure is likely to be an indispensable requirement for SN on grape berries too. The strong negative logarithmic correlation of FST and exposure time to heat intensity showed that a threshold temperature range might exist that leads to SN. When the exposure time exceeded 60 min, temperatures of about 46-47°C were sufficient to induce sunburn for berries previously grown in shade in an early stage of development (EL-31). Our observations during the wind experiment in Geisenheim support this idea. The time of exposure of clusters to direct sunlight *in vivo* depends on numerous biotic and abiotic factors (Smart, 1985; Gambetta et al., 2021) but as seen from **Figure 1** can last up to three hours for a defoliated fruit zone.

### Irrigation and defoliation experiment

Exposure of grape berries by leaf removal enhanced their resilience to heat damage. Leaf removal after bloom time is a common practice to prevent fungal diseases (Mundy et al., 2022), and our study demonstrated that it lowers the berries’ susceptibility to sunburn after an acclimation period of about 7 days. The same effect was shown for both early (pea size) and late (lag phase) defoliation. These results help in the decision making of growers for timing of leaf removal, since a relatively short period of acclimation can be matched with weather forecast data to perform leaf removal in a time of relatively low temperatures or low solar radiation. In the context of climate change, water stress does not necessarily lead to a higher susceptibility of grape berries to SN (Santos et al., 2020). In apple fruit, Makeredza et al. (2013) found low soil water contents linked to higher fruit surface temperatures and consequently to higher sunburn incidences at harvest. In contrast to apples, grape clusters are commonly found in the lower part of the canopy of most training systems and are often protected by one or several layers of leaves (Smart, 1985). Due to a very low number of stomata, which become dysfunctional by veraison, the bulk of grape berry transpiration occurs *via* the cuticle. Berry transpiration rate is thus mainly driven by the atmospheric vapor pressure deficit (VPD), is dependent on berry size and changes throughout development (Zhang and Keller, 2015). By removing leaves from the cluster zone, the evaporative demand was increased throughout various training systems in a study by English et al. (1990). The leaves may therefore not only provide natural shading but could also help to cool the berries by transpiration and consequently lower the VPD in the cluster zone.

Berries on water-stressed grapevines were less or similarly susceptible to SN compared to fully irrigated vines, giving first evidence of a drought priming induced heat tolerance in grapevine. This phenomenon has been observed in several other species, mainly in field crops (Zhang and Huang, 2020). Drought induced priming was more evident in the final SN assessment, where exposed bunches (eastern side) from the FI treatment showed higher damage than those in the drought stressed treatment. The LT_50_ of drought stressed vines in the heating experiment was higher only 3 out of 5 comparisons than that of FI vines only in, yielding rather inconclusive results.

Although daily maximum temperatures exceeded 40°C on several days in late July, sunburn severity in the 2022 growing season was generally low in our field trial and rarely exceeded 2% total damage. Our findings show that clusters under FI were more or similarly susceptible to SN when defoliation was applied on the east side of the canopy. No defoliation was conducted on the west side of the canopy. Nevertheless, the damage on the west side of the DS treatment was comparable to that on the defoliated east side of the FI treatment.

Depending on the trellis system and the overall growth, a more porous canopy structure of vines under DS may lead to a higher percentage of sunlight-exposed berries (Keller et al., 2016). Additionally, drought stress reduces the leaf area growth of a canopy and can alter leaf angles (Briglia et al., 2020). Compared with FI vines, the vines in the DS treatment had lower vigor due to water stress (-1.2 MPa), which limited the development of foliage that might provide shade to the fruit and exposed berries on the west side of the canopy to high temperatures in the afternoon (**Figure 2**). Consequently, exposed berries might accumulate more light-dependent metabolites that protect the berries (Gambetta et al., 2020) but in turn are more likely to suffer sunburn injury than shaded berries, since higher surface temperatures are more likely to be reached in sun-exposed berries. This is especially true for the west side of a north-south orientated vineyard, where foliage manipulation should be carried out with particular care to minimize heat damage on clusters.

### Wind experiment

Wind reduces sunburn incidence and severity. In order to highlight the importance of wind in the surrounding of the bunches, we have deliberately chosen two extremes between a sunburn-sensitive grape variety and a less susceptible variety. The results of this case study showed two opposing reactions which was observed in two distinct grape cultivars (Riesling and Calardis Blanc), although sunburn damage was negligible in the latter, confirming field observations at the breeding station (JKI, 2023). These results clearly show that extreme berry temperatures (in this case roughly > 48°C for Riesling) cause sunburn, especially when clusters are suddenly exposed to heat. Wind velocities applied in our study closely resemble meteorological conditions found in the area, with the closest weather station (~300 m from the experimental site) showing mean wind velocities of 2 m s^-1^ for the afternoon hours of July 2022 (14:00 – 18:00 CET) at 2 m above ground. Consequently, wind is an important factor in predicting sunburn formation and should be carefully considered when modelling sunburn formation. From a practical point of view, under similar ambient temperatures wind-exposed vineyards will have a reduced risk of sunburn formation due to lower peak berry temperatures. This makes such vineyards better suited for the application of canopy management techniques such as leaf removal, which might also have implications for berry quality and disease control. Mean average cluster temperatures in this experiment did not exceed 43.5°C, only about 5.5°C above ambient temperature, perhaps because the presence of shaded clusters had a buffering effect on the mean cluster temperatures. Maximum berry temperatures reached more than 53°C, which were limited to a few compact clusters with surfaces perpendicular to the sun zenith. These observations highlight the variability of cluster temperatures in grapevine canopies even after leaf removal. They also explain why sunburn damage in temperate climates is often localized to the most exposed parts of the most exposed clusters in a canopy, while damage is more widespread in warm climates.

## Conclusion

Our study provides new insights into the main abiotic factors that alter grape susceptibility to sunburn. Cooling effects through wind can be considered as a crucial parameter to lessen sunburn damage. Higher wind velocities were shown to reduce heat damage on susceptible Riesling grapes by reducing the fruit surface temperature through forced convection. The duration of berries being exposed to heat had a pronounced effect on the probability of sunburn development, with longer exposures decreasing the temperature required to induce damage. Berries grown under water stress conditions were less or equally susceptible to sunburn than berries on fully irrigated vines at two pre-veraison developmental stages. Defoliation and subsequent adaptation of berries through sunlight exposure within about 7 days had a more pronounced effect on the susceptibility to sunburn compared to water stress. These findings support growers in decision making concerning row orientation, water management, and timing of leaf removal.

## Data availability statement

The raw data supporting the conclusions of this article will be made available by the authors, without undue reservation.

## Author contributions

KM, MK, MS and MF conceived and planned this study with equal contributions, KM, MK and MF conducted the experiments and carried out the measurements. KM and MF drafted the original manuscript and finalized the manuscript. MK and MS reviewed and edited the manuscript. All authors approved the manuscript for publication.
