# Supplementary material for: Wind speed, sun exposure and water status alter sunburn susceptibility of grape berries
Source: Front Plant Sci. 2023 Mar 27;14:1145274. doi: 10.3389/fpls.2023.1145274 (PMC10083509; doi:10.3389/fpls.2023.1145274)
Supplement: Supplementary file 1 [file Table_1.docx]

# Supplementary Data:

## Suppl.- Data 1:

|  | 07:00 to 10:00 | | 10:00 to 13:00 | | 13:00 to 16:00 | | 16:00 19:00 | |
| --- | --- | --- | --- | --- | --- | --- | --- | --- |
|  | east | west | east | west | east | west | east | west |
| Defoliated | 34.5 | 30.7 | 38.8 | 38.2 | 38.8 | 44.7 | 39.4 | 44.3 |
| Not Defoliated | 33.0 | 29.8 | 40.5 | 37.5 | 43.2 | 43.6 | 41.6 | 46.1 |

Suppl. Data 1: Heat map of mean temperatures (°C) measured in 3-hour intervals with 2 measurements per hour by dataloggers placed at fruit height on the east and west side of the canopy of two defoliation treatments from DOY 208 - 212. Leaf removal was exclusively performed on the east side of the canopy.

## Suppl. Data 2:


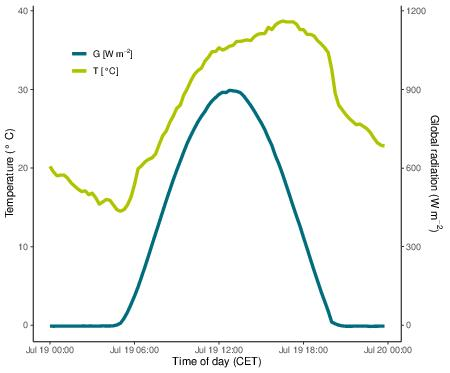


Suppl. Data 2: Air temperature and global radiation during 19 July of the wind experiment in a vineyard at Geisenheim, Germany.
